# Editorial: DNA damage and repair in reproductive and embryo cells

**DOI:** 10.3389/fcell.2023.1274341

**Published:** 2023-08-29

**Authors:** Lucia Rocco, Filomena Mottola, Shubhadeep Roychoudhury

**Affiliations:** ^1^ Department of Environmental, Biological and Pharmaceutical Sciences and Technologies, University of Campania Luigi Vanvitelli, Caserta, Italy; ^2^ Department of Life Science and Bioinformatics, Assam University, Silchar, India

**Keywords:** oxidative stress, genotoxicity, antigenotoxicity, antioxidant molecules, reproductive diseases, oxidative damage, DNA-damage responses, antioxidant therapies

Oxidative stress (OS) is a condition that happens when oxidants overwhelm the antioxidant protection systems of cells. Several studies have confirmed the production of a considerable range of oxidants by different cell types. They are usually indicated as reactive oxygen species (ROS).

Oxidizing species easily react with any adjacent molecules including biological macromolecules such as unsaturated lipids, proteins and DNA, thereby impairing normal cellular activities. OS has been associated with various diseases such as cardiovascular pathologies, brain disorders, diabetes, as well as female and male infertility.

Several factors, such as lifestyle, exposure both to environmental contaminant and cytotoxic and genotoxic substances, can cause an imbalance between ROS production and antioxidants, inducing maturational competency compromise, DNA fragmentation, apoptosis, and consequent reduction in the seminal fluid quality. During embryogenesis, increased levels of ROS are occasionally required to promote a specific stage advancement, while persistently levels of ROS impair embryo development and reduce the live birth rate.

This Research Topic received four articles aiming to elucidate the role of OS in the onset of reproductive diseases and altered embryo development as well as to clarify the importance of supplementation of antioxidant substances to trigger damage repair molecular pathways.

This Research Topic includes two reviews. In recent decades, studies have raised the interest for research on the biological role of the Y chromosome in other functions beyond the reproductive tract. For example, it has been shown that the Y chromosome can regulate gene expression, immune function, and response to OS. Xu and Pang introduce a discuss on the structure and repetitive sequences of the Y chromosome. They also summarize the correlation between Y chromosome deletions and male infertility, and provide a research perspective for further exploration of the molecular mechanism of Y-deletion and male infertility.

In conclusion, they consider the role of the Y chromosome genes or sequences in the regulatory network from the perspective of a dynamic process. Through technological innovation, the study of the repeat sequences can provide detailed and illuminating information to assess their function. To implement strategies for diagnosis, prevention and treatment of diseases, it is also necessary to know the gene members involved in their pathogenesis and understand their role.

Evidence on the reproductive consequences of human exposure to endocrine disrupting chemicals (EDCs) is sparse and/or conflicting in the scientific literature. In their review article, Dutta et al. provide a comprehensive overview of studies emphasizing the combined toxicity of EDCs on human reproduction. After acute or chronic exposures to combinations of EDCs, increased OS, elevated antioxidant enzymatic activity, disrupted reproductive cycle, and reduced steroidogenesis are often reported. The article also discusses the concentration addition (CA) and independent action (IA) prediction models, which reveal the importance of synergistic actions of EDCs mixtures. More crucially, this evidence-based study addresses the research limitations and information gaps, as well as particularly presents the future research views on combined EDCs toxicity on human reproduction.

Two original research articles complete this special issue. In their article, Bakare et al. utilize the mouse sperm morphology assay to evaluate the reproductive genotoxicity of four first-line standard anti-tubercolosis drugs (RIF, INH, EMB, PZA), and their fixed-dose combination (FDC). Histological examination of mice whose testes were exposed to these drugs is also presented. Evidence shows an increase in abnormal spermatozoa following exposure to different doses of each drug and a dose-dependent decrease in the frequency of morphologically abnormal spermatozoa after exposure to FDC. The specific reason for the increasing frequency of abnormal sperm cells is not apparent, and opinions differ on this matter. Minor modifications in testicular DNA and point mutations occur during the arrangement of DNA in the sperm head and have been proposed as possible genetic causes of abnormality in spermatozoa. The histological and genotoxic changes in the testicular cells of the treated mice could have been due to the ability of metabolites of the anti-TB medications to cross the blood testes barrier. Besides, as regards the possible action of each of the individual drugs and their metabolites, OS may also be part of the causative process.

In their research article, Vyas et al. investigate the effect of ethanolic extract of *C. borivilianum*root (CRE) on cauda epididymal spermatozoa of *Mus musculus* protection after gamma irradiation. They depict a protective role of CRE in maintaining sperm parameters. Based on the results of this study, CRE protects sperm during the process of epididymal maturation from OS induced damage by ionizing radiation. The authors conclude that CRE shows remarkable antioxidative potential, and that the extract provides improved tolerance against oxidative damages during both spermatogenesis and sperm maturation.

We hope that the Research Topic and areas covered in it will attract readers from the scientific community to help to increase our knowledge on reproductive mechanisms and fertility.

